# Infantile Onset of Spinocerebellar Ataxia Type 5 (SCA-5) in a 6 Month Old with Ataxic Cerebral Palsy

**DOI:** 10.1007/s12311-019-01085-7

**Published:** 2019-11-12

**Authors:** Gillian Rea, Sandya Tirupathi, Jonathan Williams, Penny Clouston, Patrick J. Morrison

**Affiliations:** 1 Departments of Genetic Medicine, Regional Genetics Centre, Belfast Health and Social Care Trust, A Floor, Belfast HSC Trust, Belfast, BT9 7AB UK; 2 Paediatric Neurology, Belfast Health and Social Care Trust, Belfast, BT9 7AB UK; 3 Oxford Regional Genetics Laboratories, Churchill Hospital, Oxford, OX3 7LE UK; 4 Centre for Cancer Research and Cell Biology, Queens University Belfast, Belfast, BT9 7AE UK

**Keywords:** Spinocerebellar ataxia, Cerebral palsy, Ataxic cerebral palsy

## Abstract

Spinocerebellar ataxia type 5 (SCA-5) is a predominantly slowly progressive adult onset ataxia. We describe a child with a presentation of ataxic cerebral palsy (CP) and developmental delay at 6 months of age. Genetic testing confirmed a c.812C>T p.(Thr271Ile) mutation within the *SPTBN2* gene. Seven previous cases of infantile onset SCA-5 are reported in the literature, four of which had a CP presentation. Early onset of SCA-5 presents with ataxic CP and is a rare cause of cerebral palsy.

## Introduction

Spinocerebellar ataxia type 5 (SCA5) is characterized by a slowly progressive ataxia mainly affecting the cerebellum [1]. The mean age at onset is 33 years and is predominantly an adult onset ataxia. Cases have presented as early as 5 years with ataxia. Seven infantile onset cases have been described of which four have a predominantly ataxic cerebral palsy phenotype - three with the same mutation Arg480Trp within the *SPTBN2* gene (NM_006946). We delineate the phenotype of a case with SCA-5 with onset of ataxic cerebral palsy diagnosed with developmental delay at six months old.

## Patient case study

**Fig. 1:**
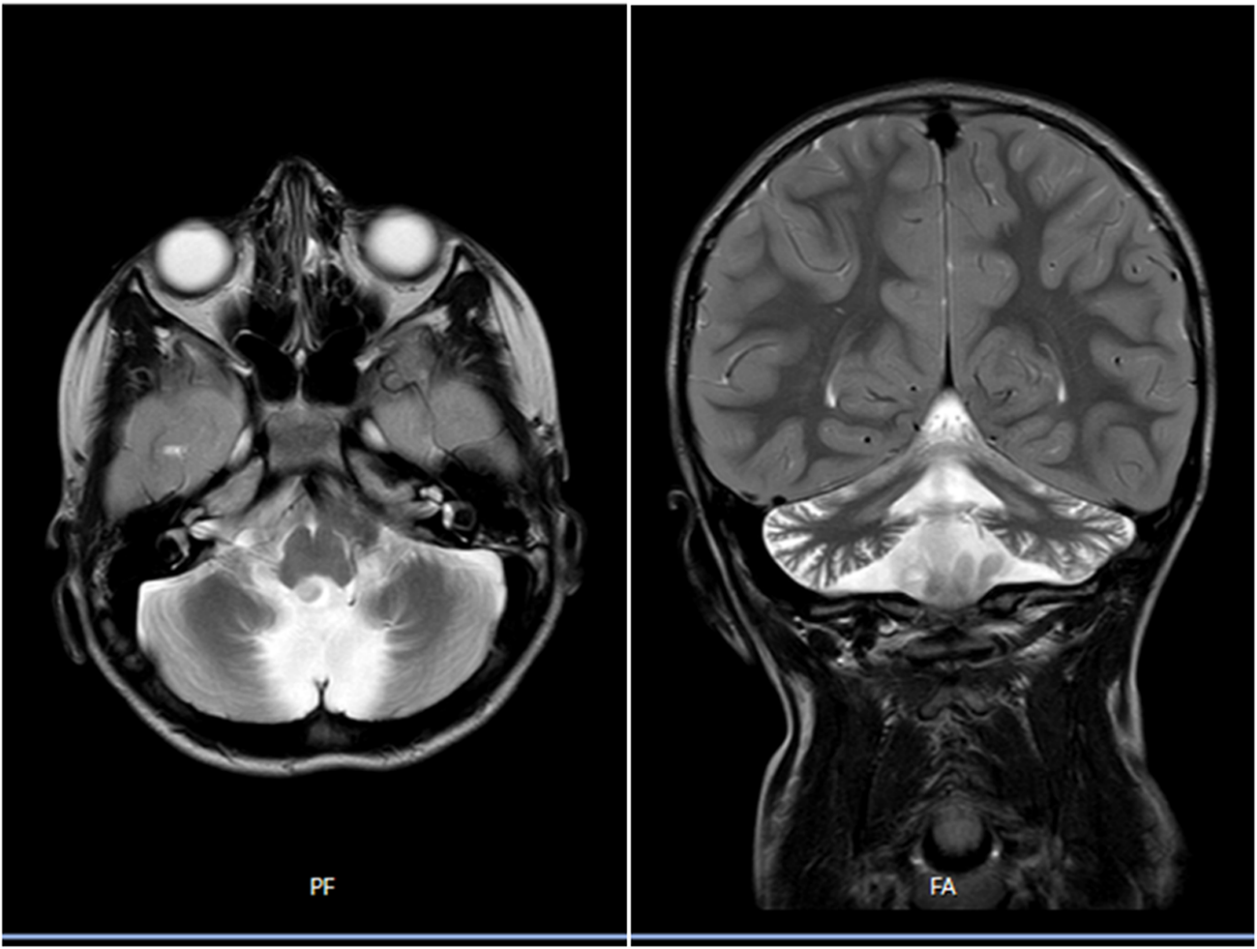
Brain magnetic resonance imaging (MR) sections at 8 years of age with coronal and sagittal midline change showing marked cerebellar atrophy

A male infant was delivered by emergency caesarean section at 33 weeks due to placental abruption. There was no family history of note. He had significant developmental delay compared to his two male siblings and rolled at six months but was unsteady on his feet and was given a label of ataxic cerebral palsy with increased reflexes and peripheral tone. He attempted to stand at one year and eventually walked at three years with a stiff gait. He had swallowing difficulties in infancy particularly for liquids, and these were still evident on video fluoroscopy at two years but slowly resolved. Blood investigations including biochemistry and CSF neurotransmitters were normal. On examination aged seven he had cerebellar ataxia with intention tremor, ataxic upper limb movements, truncal ataxia and head titubation, and clasp-knife rigidity of the lower limbs, but no nystagmus. MRI brain at one year was unremarkable but at seven years showed mild cerebellar atrophy which had progressed in the vermis at eight years (figure 1) but no evidence of brainstem, basal ganglia or other brain abnormality. Muscle biopsy was normal. He has limited speech (started to speak at 4.5 years), can hold a pencil and write his name. Genetic testing for spinocerebellar ataxia testing was normal for SCA1,2,3,6,7 and 17, Friedreich ataxia and array cytogenetic analysis. A next generation sequencing 98 gene ataxia panel confirmed a c.812C>T p.(Thr271Ile) mutation within the *SPTBN2* gene. Parental testing was normal for the c.812C>T mutation confirming a de novo origin.

## Discussion

Three cases with the same c.1438C>T p.(Arg480Trp) mutation have been described with infantile onset ataxia [2–4]. All three cases appear to be de novo mutations and occur within the second spectrin domain within the *SPTBN2* gene. In the report by Jacob et al, a girl with congenital onset of SCA5, symptoms included hypotonia, global developmental delay, walking with a wide-based gait beginning at age 3 to 4 years, intention tremor, mild dysarthria, nystagmus, facial myokymia, dysmetria, dysdiadochokinesis, hyperreflexia, and ankle clonus [2]. She was very social and studying in a normal school with a modified program at age of 12 years. In the report by Parolin Schnekenberg et al, in another girl with infantile onset of SCA5, the symptoms included head nodding and unsteady arm movements in infancy, delayed development with intellectual disability, convergent squint, poor expressive language, and ataxic gait, although she could still take a few steps with help at age of five years [3]. In the report by Nuovo et al, a two year old girl had developmental delay with ataxia and dysarthria. MRI brain showed cerebellar hypoplasia [4]. All three cases resemble the phenotype of SCAR14, an autosomal recessive ataxia due to homozygous mutations within the *SPTBN2* gene. Nicita et al [5] described four cases, two mutations c.479T>C p.(Phe160Cys) and c.185C>T p.(Thr62Ile) within the CH-1 domain and c.1309C>T p.(Arg437Trp) and c.1310G>A p.(Arg437Gln) within the second spectrin domain, all with onset under 10 months with the Arg437Trp case having a similar ataxic CP presentation to our case. Five cases of SCAR14 have been described all with homozygous mutations within *SPTBN2* and congenital onset of ataxia in normal parents. Our case has a mutation within the calpain homology (CH) domain 2 of SPTBN2 – the only other known mutation within the CH-2 domain is a German family with later onset SCA-5 with a c.758T>C mutation p.(Leu253Pro) [6]. It is thought that the Arg480Trp mutations act on the spectrin protein folding and a similar effect may be caused by the mutation in our case as mutations within the CH-2 domain in drosophila alter mobility and recruitment of β-III-spectrin and cause loss of dendritic arborisation within the developing cerebellum. Our case along with the Arg437Gln and the three Arg480Trp cases all present with the common phenotype of developmental delay in early infancy with progressive hyperreflexia, all developing into ataxia with intellectual disability with imaging findings of cerebellar hypoplasia or atrophy (Table 1). It will be interesting to see if any further identified mutations within the CH-2 domain suggest a gradient effect with more severe dendritic arborisation loss with more 3’ mutations. We note that the German family with SCA-5 mutation within the CH-2 domain also exhibited tremor, a finding not prominent in the other cases of adult onset SCA-5 but evident in our case by age 7, and the Arg437Gln case (age of onset not specified but somewhere between onset of ataxia at 5 months and examination at 18 years so tremor may be a later presenting feature).

**Table 1:** Features of early onset SCA-5 cases

Patient	Current case	Jacob [2]	Parolin-Schnekenberg [3]	Nuovo [4]	Nicita [5]			
Sex	M	F	F	F	M	F	F	M
Age at onset	6 months	12 months	8 months	22 months	5 months	8 months	10 months	5 months
Ethnicity	Caucasian	Caucasian	‘Mediterranean’	n/k	n/k	n/k	n/k	n/k
Variant	c.812C>T	c.1438C>T	c.1438C>T	c.1438C>T	c.479C>T	c.185C>T	c.1309C>T	c.1310C>A
Protein	T27I	R480W	R480W	R480W	F160C	T62I	R437W	R437Q
Inheritance	*de novo*	n/k	*de novo*	*de novo*	*de novo*	n/k	*de novo*	*de novo*
1^st^ symptom	Ataxic gait	Ataxic gait	Dev del	dysarthria	Dev del	Dev del	Dev del	hypotonia
Cerebellar findings	Ataxia	ataxia	Ataxia	ataxia	Ataxia	ataxia	Ataxia	ataxia
Ocular nerve	-	nystagmus	strabismus	nystagmus	strabismus	nystagmus	-	nystagmus
Pyramidal signs	brisk reflexes	brisk reflexes	N	N	-	-	-	brisk reflexes
Tremor	-	+	-	+	-	-	-	+
Dystonia	+	-	-	-	-	-	-	-
Developmental Delay	+	+	+	+	+	+	+	+

+ present; - absent; NK not known; M male; F female; dev del developmental delay; N normal

Any rare heterozygous mutation within the *SPTBN2* gene sheds light on mechanisms causing both SCA-5 and SCAR14 as the infantile phenotype is similar in both disorders, but the adult SCA-5 phenotype is different. It is likely that further early onset cases will be identified and clinicians should consider both SCA-5 and SCAR14 as rare causes of cases of infantile ataxia or cerebral palsy. All infantile cases to date have not had a family history of ataxia.

## Conclusion

Early onset of SCA-5 may present with an ataxic CP phenotype and is a rare cause of cerebral palsy. Clinicians should be aware of potential genetic aetiologies for cases of non progressive congenital or early onset ataxia with CP and consider next generation panel testing or exome sequencing for such cases.
